# Efficacy of *Thermus thermophilus* Fermentation Extract in Promoting Hair Growth and Improving Scalp Health in Androgenic Alopecia: A Clinical and In Vitro Study

**DOI:** 10.1111/jocd.70095

**Published:** 2025-03-06

**Authors:** Yudong Hu, Zeyu Wei, Xusheng Wu, Miaojuan Peng, Guang Sun, Qingmei Liu, Qian Liu, Sophia Yi Zhang

**Affiliations:** ^1^ Yatsen Global Innovation R&D Center Shanghai China; ^2^ Department of Dermatology, Huashan Hospital, Shanghai Institute of Dermatology Fudan University Shanghai China; ^3^ Guangzhou Yatsen Global Co. Ltd Guangzhou China; ^4^ School of Life Sciences Zhejiang Chinese Medical University Hangzhou China

**Keywords:** AGA, anti‐inflammation, hair loss, TTFE

## Abstract

**Background:**

Androgenic alopecia (AGA) is a widespread form of hair loss characterized by the gradual reduction in follicular size and reduced hair density, with unsatisfactory treatments.

**Aims:**

This study explores the therapeutic potential of a novel fermented extract, *Thermus thermophilus* fermentation extract (TTFE), in mitigating the effects of AGA.

**Methods:**

The research integrates in vitro assays utilizing various hair‐growth‐related cells with a 12‐week clinical trial involving 32 male subjects diagnosed with AGA. A 12‐week clinical trial involving 32 male subjects diagnosed with AGA evaluated the effects of topical TTFE application on scalp hydration, transepidermal water loss (TEWL), sebum levels, and hair density using a hydration pin probe, tewameter, and sebumeter and a dermoscope respectively. Moreover, in vitro studies analyzed TTFE's effects on cell proliferation, ATP production, inflammation, key signaling pathways.

**Results:**

The study revealed that the topical administration of TTFE increased hair density (96.88%), follicular diameter (15%), and scalp hydration while reducing TEWL (47.43%) and sebum production (20%). The in vitro results corroborated these findings, showing that TTFE significantly enhanced hDPCs proliferation by increased ATP production and Ki67 expression, and upregulated β‐catenin and downregulated TGF‐β2. Additionally, TTFE demonstrated strong anti‐inflammation by suppressing the proinflammatory cytokines IL‐1β and IL‐6 with decreased ROS.

**Conclusions:**

TTFE promotes hair growth and scalp health by improving follicles, modulating Wnt/β‐catenin and TGF‐β signaling, and reducing inflammation. These findings suggest TTFE holds promise as a potential treatment for AGA, offering a multifaceted approach to restoring hair growth and scalp health by targeting both follicular biology and inflammation.

## 1 Introduction

Hair growth follows a cyclical process consisting primarily of three distinct phases: anagen (growth), catagen (transition), and telogen (rest) [1]. The anagen phase, lasting several years, drives hair elongation and is regulated by key pathways such as Wnt/β‐catenin, promoting follicle stem cell proliferation [2]. Catagen is a brief phase marked by apoptosis, primarily governed by TGF‐β signaling, which can trigger premature follicle regression [3]. During the telogen phase, follicles remain dormant for months before re‐entering anagen [4]. Hair loss treatments aim to prolong the anagen phase and reduce catagen and telogen durations. AGA, commonly referred to as male or female pattern baldness, represents the most widespread form of hair loss, driven predominantly by genetic predispositions and the androgen hormone dihydrotestosterone (DHT), which binds to androgen receptors (AR) in hair follicles, resulting in shorter duration of the anagen (growth) phase and leading to their miniaturization and a subsequent decline in both hair density and thickness [5]. Although it is not a life‐threatening issue, the influence of AGA extends beyond the physical, significantly affecting psychological well‐being [6].

Pharmacological treatments include minoxidil, a potassium channel opener [7], and finasteride, a 5α‐reductase inhibitor that lowers DHT [8], though both have side effects like irritation or sexual dysfunction [9]. Despite the availability of these treatment options, these treatments do not provide satisfactory results for AGA, highlighting the need for alternative therapies with improved safety and efficacy profiles. *Thermus thermophilus* ( *T. thermophilus* ) was first isolated from a hot spring in Japan and is known for its extremophilic proteins, which remain active and stable at temperatures above 80°C [10]. Due to its high polyamine content, such as spermidine and spermine, *T. thermophilus* extracts have been shown to extend lifespan across species and delay skin aging by promoting autophagy [11].

This study assesses the efficacy of a novel topical formulation containing TTFE, a novel fermented extract from *T. thermophilus* with significant hair growth‐promoting and anti‐inflammatory properties, in enhancing hair follicle proliferation and reducing hair loss in individuals with AGA. This study seeks to elucidate the potential of TTFE as a therapeutic agent for AGA.

## 2 Material and Methods

### 2.1 Reagents

TTFE was supported by Hangzhou Youmada Biotechnology Co. Ltd. Minoxidil, DMSO, MTT, and Dihydrotestosterone (DHT) were provided by Sigma‐Aldrich. RNAiso Plus, PrimeScript RT reagent Kit, SYBR Premix Ex Taq II, and sterile ddH_2_O were purchased from Takara Biomedical Technology (Beijing) Co. Ltd. Human IL‐1β and IL‐6 ELISA kits were obtained from Abcam. Reactive oxygen species (ROS) detection and ATP production kits were bought from Beyotime Biotechnology.

### 2.2 MTT Assay

The MTT assay was conducted to assess the effects of TTFE on cell viability and proliferation. Briefly, cells were placed into 96‐well plates and incubated overnight. Subsequently, cells were stimulated with various indicated concentrations (0%–20%) of TTFE for 24 h, and the final volume of medium in each well was adjusted to 200 μL, with 10% DMSO as the positive control (PC) and the solvent as the negative control (NC). Following treatment, the medium was removed, and MTT solution (0.5 mg/mL) was added to each well. After 4 h of incubation at 37°C, the medium was replaced with 150 μL DMSO to solubilize the formazan crystals. Absorbance was measured at 490 nm using a microplate reader. For cell proliferation assay, human dermal papilla cells (hDPCs) were plated into 96‐well plates and allowed to attach overnight. On the following day, 800 nM DHT was used to stimulate the cells and then different concentrations of TTFE were added into wells for different time points. Afterward, the detection process was consistent with the method of detecting cell viability using the MTT reagent.

### 2.3 Enzyme‐Linked Immunosorbent Assay (ELISA) Assay

A total of 1.1 × 10^4^ hDPCs were dispensed into 6‐well plates and incubated overnight until cell confluence reached 50% ~ 60%. Subsequently, cells were stimulated with 0.3125% TTFE with or without 800 nM DHT for 24 h (minoxidil was used as PC), and the supernatant from each well was then harvested for the subsequent ELISA test according to the corresponding guidelines. In brief, 50 μL of each sample or standard buffer was pipetted into the designated wells, followed by the introduction of 50 μL of the antibody mixture. The plates were subsequently cultivated for 2 h at ambient temperature on a plate shaker. Following incubation, the plates were rinsed three times with washing buffer, and 100 μL of the TMB substrate solution was introduced into each well. The plates were then incubated for 10 min in darkness. Finally, 100 μL of stop solution was incorporated into each well, mixed for 1 min with shaking, and the optical density (OD) at 450 nm was measured.

### 2.4 Quantitative Real‐Time PCR (qPCR) Assay

A total of 1.1 × 10^4^ hDPCs were cultured into 6‐well plates and stimulated the following day with 0.3125% TTFE for 24 h, either in the presence or absence of 800 nM DHT, with minoxidil serving as the PC. The cells were subsequently rinsed twice with pre‐chilled PBS, after which 1 mL of RNAiso Plus was introduced into each well for cell lysis. Following the manufacturer's instructions, mRNA was then isolated using an mRNA isolation kit and then the mRNA concentration was quantified using a NanoDrop spectrophotometer, followed by reverse transcription into cDNA using the cDNA Reverse Transcription Kit. Finally, the qPCR procedure was performed with specific primers (Table 1) purchased from Sangon Biotech, and data analysis was conducted using the 2^−ΔΔCT^ method to quantify changes in mRNA levels.

**TABLE 1:** Primers with nucleotide codes used for qPCR analysis.

Gene name	Sequence (5′ – > 3′)
GAPDH	Forward primer: GGAGCGAGATCCCTCCAAAAT
	Reverse primer: GGCTGTTGTCATACTTCTCATGG
TGF‐β2	Forward primer: CAGCACACTCGATATGGACCA
	Reverse primer: CCTCGGGCTCAGGATAGTCT
β‐catenin	Forward primer: AAAGCGGCTGTTAGTCACTGG
	Reverse primer: CGAGTCATTGCATACTGTCCAT
DKK1	Forward primer: CCTTGAACTCGGTTCTCAATTCC
	Reverse primer: CAATGGTCTGGTACTTATTCCCG

### 2.5 ATP Production Detection Assay

hDPCs were inoculated into 6‐well plates and kept overnight under incubation conditions until reaching approximately 60% confluency. hDPCs were seeded into 6‐well plates and incubated overnight until cell confluency reached 60%. Cells were treated with 800 nM DHT with or without 0.3125% TTFE and 500 μM minoxidil served as PC. After 24 h, cells were washed twice with PBS and then 200 μL lysis buffer was dispensed into each well, followed by the collection of the cell lysis. Cell lysates were used to detect the ATP production according to the manufacturer's instructions. Briefly, 20 μL of cell lysate was homogenized with 100 μL of ATP detection working solution, and the mixture was mixed quickly and thoroughly before fluorescence intensity was measured by luminometer.

### 2.6 In Vitro Hair Follicle Staining Assay

The isolated hair follicles were placed on a culture dish and washed twice with PBS containing antibiotics (including 200 units/mL penicillin and streptomycin) and then transferred to a new glass dish and separated into single pieces with a length of 3–4 mm using sterile tweezers and scissors. Afterwards, the separated hair follicles were placed in 500 μL of culture medium in 24‐well plates at 37°C under 5% CO_2_. After 24 h, the hair follicles were observed under an inverted microscope, and growing hair follicles were selected for the following experiments. Subsequently, 500 μL of culture medium was applied to each well in the blank control group. For the PC group, 500 μL of culture medium containing 10 μg/mL insulin and 10 ng/mL hydrocortisone was incorporated into each well. In the sample group, 500 μL of culture medium containing 0.3125% TTFE was introduced. Then the plates were placed under incubation conditions for 120 h, with the medium replaced every other day. Finally, the hair follicles were harvested, fixed in 4% paraformaldehyde, embedded in paraffin, and sectioned for the following immunofluorescence detection with Ki67 antibody. Images were captured using a microscope.

### 2.7 Reactive Oxygen Species Assay

HaCaT cells were dispensed into 12‐well plates at a density of 1.5 × 10^5^ cells/well and maintained overnight under incubation conditions. The following day, the cells were exposed to 0.3125% TTFE for 22 h, followed by exposure to 2 mM H₂O₂ for an additional 2 h. The cells were then harvested and resuspended in a DCFH‐DA solution (diluted 1:1000 in serum‐free culture medium to a final concentration of 10 μM). During incubation at 37°C for 20 min, the suspension was gently inverted every 3–5 min to ensure proper mixing. Subsequently, the cells were washed three times with serum‐free culture medium to completely remove any unbound DCFH‐DA probe. The fluorescence intensity at Ex: 488 nm and Em: 525 nm was detected using flow cytometry.

### 2.8 Clinical Study

32 subjects with AGA and Hamilton–Norwood rating ≥ 3 or Sinclair rating ≥ 2 were recruited and the effect of the formulation on promoting hair growth was evaluated by comparing the results before and after application. For the formulation usage, the subjects were required to apply the formulation containing TTFE evenly to the scalp/hair roots and massaged appropriately for 1–2 min until fully absorbed. At the indicated time points, hydration pin probe, tewameter, and sebumeter probes were used to accurately measure changes in physiological parameters such as hydration (μS), transepidermal water loss (TEWL, g/h/m^2^), and sebum (μg/cm^2^) in the shaved scalp area of the subjects. Meanwhile, a dermoscope was used to take images of the shaved scalp area of the subjects and analyze changes in hair‐related indicators, including the diameter, number, and density of hair shafts, as well as the number and proportion of various types of hair follicle units. The subjects included in this study were all males from Shanghai who had no skin diseases or other major diseases in the past 3 months and did not take any drugs, antibiotics, or hormones that affect or promote hair growth. Formal written consent was obtained from all subjects prior to the study, with each participant signing and confirming their consent after thorough communication. The study plan was approved by the Medical Ethics Committee of Shanghai Jing'an District Central Hospital (approval number AF/SC‐11/02.0) on April 12, 2023, and was strictly supervised and guided by the Ethics Committee throughout its duration. This study strictly protects the personal privacy information security and data confidentiality of the subjects.

### 2.9 Quantification and Statistical Analysis

Statistical analyses were conducted using GraphPad Prism (version 8/9), and data are presented as mean ± standard deviation (SD). Group comparisons were statistically assessed using the *t*‐test. Compared with the BC group, significance is represented by *, with the following thresholds: ^ns^ *p* > 0.1234, *p < 0.0332, **p < 0.0021, ***p < 0.0002, ^****^ *p* < 0.0001.

## 3 Results

### 3.1 Topical Administration of TTFE Promotes Hair Growth and Improves Scalp Health

**FIGURE 1:**
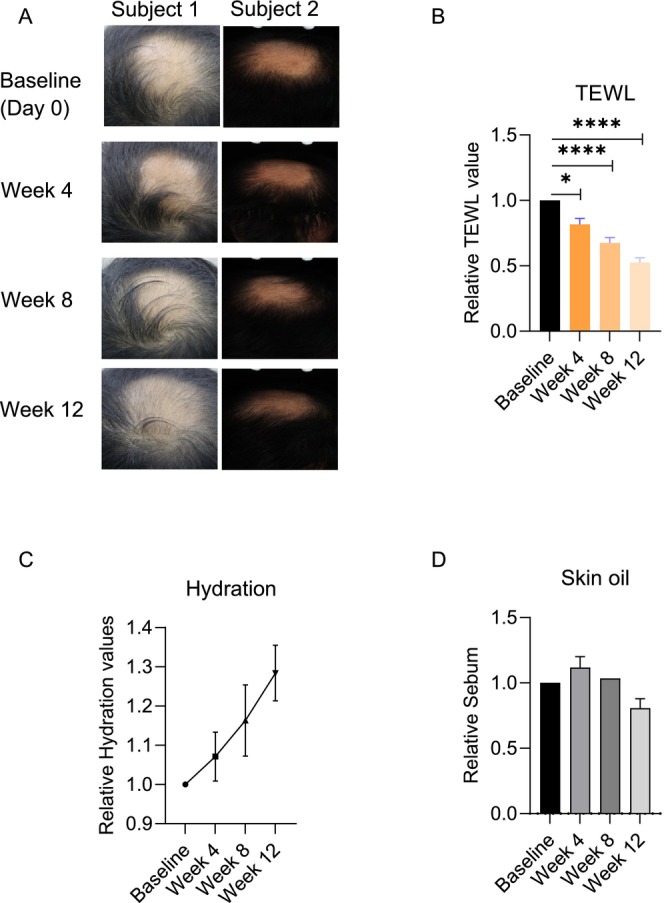
Topical administration of TTFE promotes hair growth and improves scalp health. (A) Representative pictures of subjects on baseline (day 0), week 4, week 8, and week 12. (B) Changes in TEWL values after 4, 8, and 12 weeks of TTFE use compared to that of baseline and data are shown as mean ± SD (*p < 0.0332, ^****^ *p* < 0.0001). (C) Changes in relative hydration values after 4, 8, and 12 weeks of TTFE use compared to that of baseline and data are shown as mean ± SD (^ns^ *p* > 0.1234). (D) Changes in relative sebum values after 4, 8, and 12 weeks of TTFE use compared to that of baseline and data are shown as mean ± SD (^ns^ *p* > 0.1234).

To assess the effects of TTFE on hair growth and conditions of the scalp, TTFE was topically applied to the scalps or hair roots of 32 subjects, accompanied by a 1–2 min massage until fully absorbed. On day 0 (baseline), most subjects exhibited hair loss characterized by thinning hair diameter, reduced hair density, and the presence of short, non‐pigmented vellus hairs. After TTFE application, most subjects showed a noticeable reduction in the size of hair loss areas, accompanied by increased hair density and a darker appearance, particularly by week 12. Meanwhile, the scalp areas of subjects treated with TTFE were predominantly covered with dark hair (Figure 1A).

Sebaceous gland (SG) hypertrophy, a hallmark of AGA, often results in excess sebum production, leading to increased scalp oil secretion, greasy hair, and impaired skin barrier function [12]. Next, we explored the effects of TTFE on the water content of scalp stratum corneum, TEWL, and scalp‐saturated oil content. As illustrated in Figure 1B, TTFE remarkably reduced the TEWL values by 18.39%, 32.56%, and 47.43% at weeks 4, 8, and 12, respectively, suggesting a marked improvement in skin barrier function. Moreover, after using TTFE for 4, 8, and 12 weeks, the water content of scalp stratum corneum was increased by 7.11%, 16.30%, and 28.37% (Figure 1C), although the increase was not statistically significant. Subsequently, sebum content showed a nearly 20% reduction after 12 weeks of treatment compared to baseline (Figure 1D). Collectively, these findings indicate that TTFE facilitates hair growth and restores scalp health by reducing sebum production.

### 3.2 TTFE Strengthens the Hair Shaft and Enhances Hair Follicle Proliferation

**FIGURE 2:**
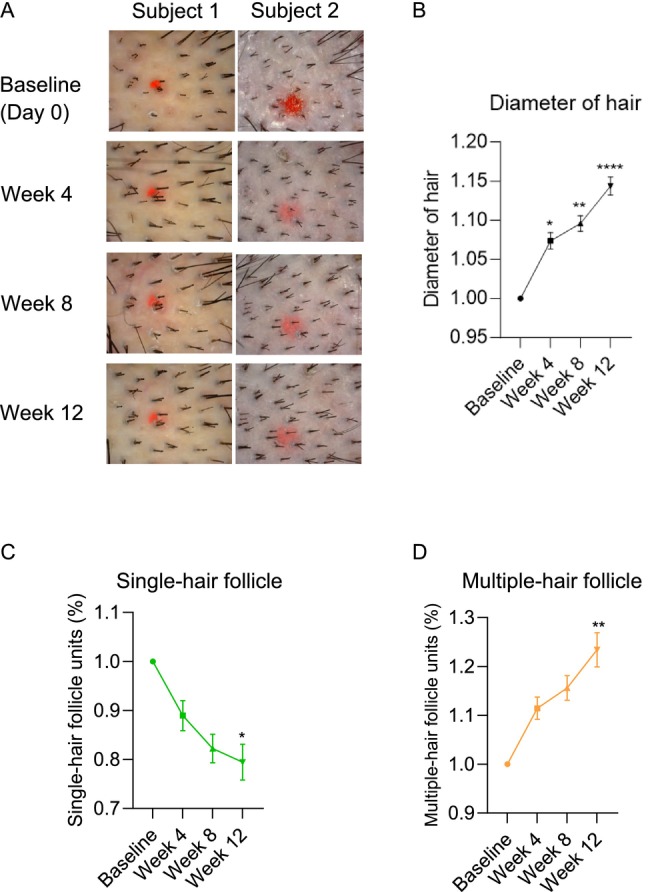
TTFE strengthens hair shafts and enhances hair follicle proliferation. (A) Representative pictures of subjects in shaved areas on baseline, week 4, week 8, and week 12. (B) Effect of TTFE on hair diameter changes after 4, 8, and 12 weeks compared to that of baseline and data are presented as mean ± SD (*p < 0.0332, **p < 0.0021, ^****^ *p* < 0.0001). (C) Change in the proportion of single hair follicle units after using TTFE for 4, 8, and 12 weeks compared to that of baseline and data are presented as mean ± SD (*p < 0.0332). (D) Change in the proportion of multiple hair follicle units after using TTFE for 4, 8, and 12 weeks compared to that of baseline. Data are shown as mean ± SD, *n* = 3 (**p < 0.0021).

To accurately investigate the effects of TTFE on hair growth, hair diameter and percentages of single‐cell and multiple‐cell follicle units were measured after depilation using a dermoscope. As illustrated in Figure 2A, macro photographs of scalp hair revealed a significant increase in hair density compared to baseline after 4, 8, and 12 weeks of using the TTFE‐containing formulation. The most pronounced effects were observed at 12 weeks, where hair appeared thicker and more abundant. Notably, 96.88% of subjects demonstrated an increase in hair density. These results suggest that TTFE significantly increases hair density after depilation.

Meanwhile, hair diameter analysis indicated that the follicles in the TTFE‐treated group exhibited a roughly 15% increase in diameter after 12 weeks of administration (Figure 2B). Since follicular units with multiple hairs (e.g., 2–4 hairs) contribute to a denser appearance, the number of hairs per follicular unit was additionally evaluated as an indicator of hair growth. As shown in Figure 2C,D, after using TTFE, the percentage of single‐hair follicle units significantly decreased by 11.05%, 17.78%, and 20.56% after 4, 8, and 12 weeks, respectively. In contrast, the proportion of multiple‐hair follicle units dramatically improved with a 23.40% increase after 12 weeks. In conclusion, these findings demonstrate that TTFE exerts a pronounced effect in promoting hair growth and maintaining hair health.

### 3.3 TTFE Exhibits Low Irritant Effects in Different Hair‐Growth‐Related Cells

**FIGURE 3:**
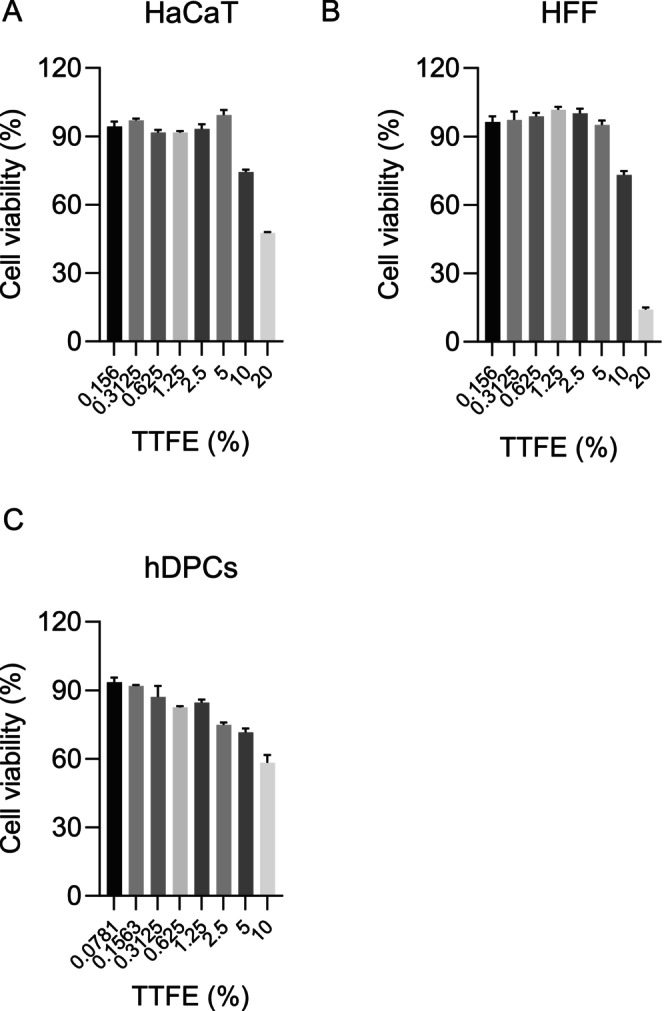
TTFE exhibits limited cytotoxicity effect in different hair‐growth‐related cells. Cells were treated with indicated concentrations (0%–20%) of TTFE for 24 h and cell viability was detected using MTT assay in human keratinocytes HaCaT (A), human fibroblasts HFF (B), and human dermal papilla cells hDPCs (C). Data are presented as mean ± SD, *n* = 3.

An MTT assay was utilized to determine the cytotoxic effects of TTFE on hDPCs, human keratinocytes (HaCaT), and human fibroblasts (HFF). Cells were exposed to different concentrations (0%–20%) of TTFE for 24 h. The results revealed that the cell viability of HaCaT and HFF cells remained above 90%, showing no decreasing trend when treated with TTFE concentrations below 5% compared to the control group. A reduction in cell viability was observed only with 10% TTFE stimulation (Figure 3A,B). These results indicate that TTFE exhibits minimal cytotoxicity in HaCaT and HFF cells. Besides, as shown in Figure 3C, TTFE incubation inhibited cell viability in hDPCs in a dose‐dependent manner. Despite this, cell viability remained above 70% even at concentrations exceeding 5%. This demonstrates that TTFE has limited cytotoxic effects in hDPCs.

### 3.4 TTFE Enhances the Proliferation of hDPCs via Promoting ATP Production

As a specialized mesenchymal cell population situated at the base of hair follicles, hDPCs are integral to dermal‐epidermal signaling processes, which regulate hair follicle activity and govern the progression of the hair growth cycle [13], thus the proliferation of hDPCs indicates the ability to promote hair morphogenesis [14]. To investigate the impact of TTFE on hDPCs proliferation, the cell proliferation rate was assessed via an MTT assay using a DHT‐induced cell model.

**FIGURE 4:**
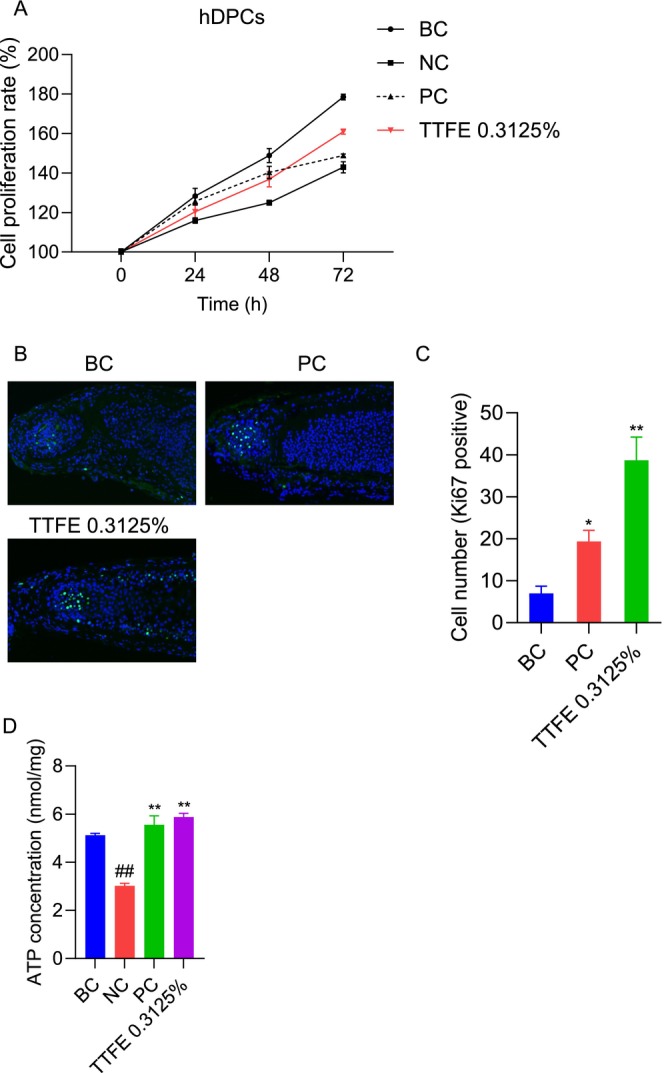
TTFE promotes the proliferation of hDPCs via upregulating ATP production. (A) The cell proliferation rate of hDPCs with 0.3125% TTFE treatment after 24, 48, and 72 h in DHT‐stimulated cells (minoxidil served as PC) and data are plotted as mean ± SD. (B) Representative immunofluorescence images showing Ki67 expression (green fluorescence) in isolated human hair follicles with insulin and hydrocortisone combination treatment serving as PC. (C) The cell number of Ki67‐positive cells was counted in each group and data are presented as mean ± SD (*p < 0.0332, **p < 0.0021). (D) Changes in ATP concentration of hDPCs with 0.3125% TTFE for 24 h. Data are shown as mean ± SD, *n* = 3 (**p < 0.0021); ##Internal reference.

As shown in Figure 4A, DHT stimulation (NC group) significantly reduced the proliferation rate of hDPCs compared to the BC control group. Noteworthy, TTFE effectively counteracted the suppression, leading to a progressive increase in hDPCs proliferation over time. After 72 h, the proliferation rate reached 160.95%, surpassing that of minoxidil (PC). To further validate the effects of TTFE on hDPCs proliferation, immunofluorescence staining for Ki67, a cellular proliferation marker, was conducted on isolated human hair follicles (HFs). Nuclei were stained with DAPI and insulin and hydrocortisone combination treatment served as PC. From Figure 4B, Ki67 expression (green fluorescence) mainly accumulated within the hair matrix beneath Auber's line. After 5 days of culture, most control (BC) HFs entered the catagen phase, exhibiting diminished proliferative ability. However, HFs treated with TTFE displayed substantially higher Ki67 fluorescence intensity than the BC group, even exceeding the PC group. Quantitative analysis revealed a notable increase in the number of Ki67‐positive cells upon TTFE treatment, indicating that TTFE promotes hair growth (Figure 4C).

Additionally, ATP production, as the powerhouse of cells, promotes cell proliferation and maintains the anagen phase of hair [15]. Since TTFE exhibited strong pro‐proliferative properties, ATP production was performed to evaluate its effects. As shown in Figure 4D, DHT treatment decreased ATP concentration, while minoxidil‐treated cells exhibited increased ATP production compared to untreated cells (BC) in the DHT‐induced cell model. Interestingly, it revealed a substantial increase in intracellular ATP production in TTFE‐treated cells, surpassing levels observed in the minoxidil‐treated (PC) group. These findings suggest that TTFE promotes hDPCs proliferation and enhances ATP production, thereby facilitating hair growth and prolonging the anagen phase.

### 3.5 TTFE Shows a Significant Anti‐Inflammatory Effect in hDPCs

**FIGURE 5:**
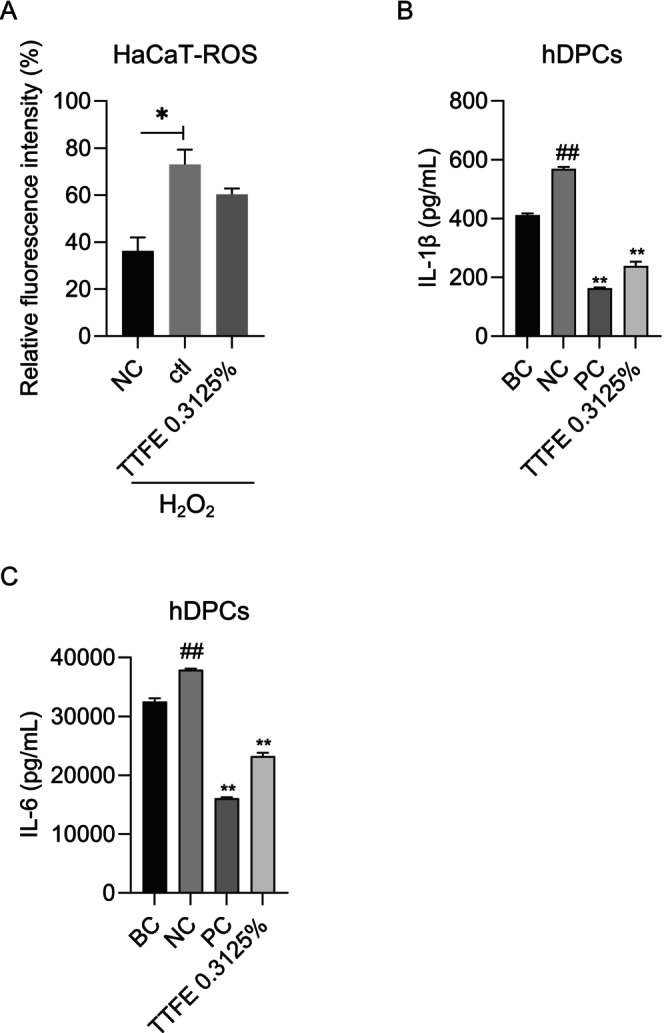
TTFE significantly inhibits inflammatory response in hDPCs via reducing ROS production. (A) Quantification of relative fluorescence intensity in TTFE‐pretreated HaCaT cells followed by H_2_O_2_ stimulation. Data are presented as mean ± SD (*p < 0.0332). (B and C) Quantification of IL‐1β concentration (B) and IL‐6 concentration (C) in DHT‐stimulated hDPCs with or without 0.3125% TTFE treatment (minoxidil served as PC). Data are shown as mean ± SD, *n* = 3 (**p < 0.0021).

Oxidative stress, driven by excessive concentrations of reactive oxygen species (ROS) or free radicals, induces oxidative damage to HFs and disrupts the hair cycle, leading to pathological hair loss [16]. A DCFH‐DA fluorescence probe was utilized to assess ROS levels. Following exposure to oxidative stress, the fluorescence intensity in the control group (ctl) was twice as high as in the negative control group (NC), indicating a significant increase in ROS production induced by H₂O₂. Notably, the fluorescence intensity in the TTFE‐treated group was reduced (Figure 5A), suggesting that TTFE may inhibit ROS production.

Moreover, it is widely accepted that ROS promotes inflammatory responses [17]. Besides, pro‐inflammatory cytokines, such as IL‐1β and IL‐6, are enhanced in the scalps of individuals with AGA, exacerbating follicular miniaturization [18]. To investigate the potential anti‐inflammatory properties of TTFE, a DHT‐stimulated hDPCs model was employed, and the levels of pro‐inflammatory cytokines (IL‐1β and IL‐6) were quantified. As shown in Figure 5B,C, DHT treatment elevated IL‐1β and IL‐6 protein levels in hDPCs, whereas TTFE significantly reduced these cytokines, mirroring effects observed with the minoxidil (PC). Collectively, these findings suggest that TTFE exerts notable anti‐inflammatory effects by inhibiting ROS production.

### 3.6 TTFE Up‐Regulates β‐Catenin and Down‐Regulates TGF‐β2 Expression in hDPCs

**FIGURE 6:**
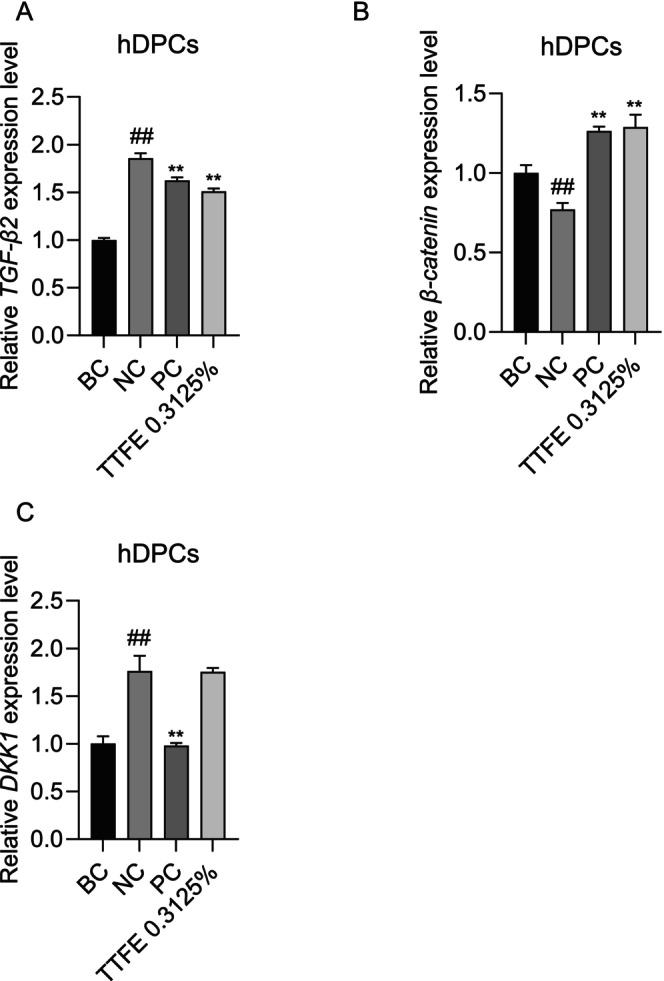
TTFE regulates Wnt and TGF‐β signaling pathways. qPCR analysis of hair growth‐related genes including *TGF‐β2* (A), *β‐catenin* (B), and *DKK1* (C) in DHT‐treated hDPCs with or without 0.3125% TTFE treatment (minoxidil served as PC), and data are plotted as mean ± SD, *n* = 3 (**p < 0.0021); ##Internal reference.

The development of hair follicles involves several signaling pathways, with the Wnt signaling and TGF‐β signaling pathways being the earliest and most critical [19]. To elucidate the potential molecular mechanisms of TTFE, its influence on TGF‐β2, β‐catenin, and DKK1 (a Wnt inhibitor) was analyzed. As depicted in Figure 6, DHT induced a significant upregulation of *TGF‐β2* and *DKK1* mRNA levels, while concurrently downregulating *β‐catenin* mRNA expression. Notably, hDPCs treated with subtoxic concentrations (0.3125%) of TTFE showed reduced *TGF‐β2* mRNA levels and increased *β‐catenin* mRNA levels compared to the DHT‐treated (NC) group. Interestingly, the impact was more noticeable than that of minoxidil.

Moreover, *DKK1*, a negative regulator of Wnt signaling that antagonizes the role of Wnt in hair growth, was measured with TTFE treatment using qPCR assay. Although minoxidil significantly inhibited the DHT‐induced up‐regulation of the *DKK1* gene level, TTFE did not exhibit a similar inhibitory effect on the *DKK1* mRNA level compared to NC group. These findings suggest that TTFE may modulate the Wnt and TGF‐β signaling pathways to promote hair growth, exhibiting effects distinct from those of minoxidil.

## 4 Discussion and Conclusion

This study highlights the potential of TTFE as a therapeutic candidate for AGA, a condition marked by progressive hair follicle miniaturization and associated scalp inflammation. The diverse effects of TTFE on hair follicle biology and scalp health suggest its promise as a potential therapeutic candidate for managing AGA through several mechanisms.

The clinical study confirmed significant improvements in hair density, diameter, overall scalp health, and a reduction in single‐hair follicle units. After 12 weeks of TTFE administration, reductions in TEWL and sebum production suggested enhanced scalp barrier function, which is typically compromised in AGA due to sebaceous gland hypertrophy and excessive sebum secretion [12]. By restoring scalp hydration and sebum balance, TTFE may create a more favorable environment for hair growth, mitigating the negative effects of excess sebum, which can lead to scalp inflammation and follicular occlusion. Additionally, the significant improvements in hair diameter and density highlight TTFE's potential to promote hair growth and strengthen existing hair shafts, which is critical for visible clinical outcomes in hair restoration. However, due to the limited diversity of populations and small sample size in the clinical study, further research is recommended to validate its efficacy and broaden its application across diverse demographic groups.

Furthermore, the in vitro results of this study corroborate the clinical findings, with one key observation being that TTFE promotes hDPCs proliferation, as evidenced by increased ATP production and Ki67 expression. hDPCs are critical for hair follicle function, regulating the transition from telogen to anagen by secreting growth factors such as Wnt and BMP [14]. By upregulating β‐catenin, a key downstream effector of the Wnt signaling pathway, TTFE promotes the transition into the anagen phase and alleviates follicular miniaturization—a primary characteristic of AGA [20]. Concurrently, the downregulation of TGF‐β2, known for inducing catagen transition and follicular apoptosis, suggests that TTFE may help prolong the anagen phase, thereby counteracting AGA effects [21]. This dual modulation of Wnt/β‐catenin and TGF‐β signaling pathways could be pivotal in restoring normal hair growth cycles in individuals with AGA. However, further evidence is needed to identify the in‐depth mechanisms.

This study also emphasizes the anti‐inflammatory properties of TTFE, demonstrated by a notable decline in proinflammatory cytokines IL‐1β and IL‐6, further enhancing its therapeutic value. Inflammation is increasingly recognized as a key contributor to follicular damage in AGA, exacerbating hair loss by disrupting hair follicle cycling and promoting fibrosis [22]. By attenuating the inflammatory response, TTFE protects hair follicles from damage and fosters an environment conducive to hair regeneration. This anti‐inflammatory action aligns with emerging therapeutic strategies targeting inflammation in AGA to improve treatment outcomes [23].

In conclusion, TTFE demonstrates potential as a multifaceted treatment for AGA by modulating key signaling pathways, exhibiting anti‐inflammatory effects, and enhancing scalp and hair health. Further studies are needed to validate its long‐term efficacy and safety in larger, diverse populations and to elucidate its molecular mechanisms. These findings support TTFE as a potential therapeutic option for AGA and other forms of hair loss.

## Author Contributions

**Sophia Yi Zhang:** conceptualization, experiments design, data curation, formal analysis, methodology, project supervision, resources, software, visualization, writing – review and editing. **Yudong Hu:** data curation, formal analysis, methodology, project administration, writing – original draft, writing – review and editing. **Zeyu Wei:** formal analysis, investigation, visualization. **Xusheng Wu:** funding acquisition, data curation, investigation, methodology, supervision. **Miaojuan Peng:** investigation, methodology, project administration. **Guang Sun:** methodology, project administration, resources. **Qingmei Liu** and **Qian Liu:** investigation, methodology. All authors have read and approved the final manuscript.

## Conflicts of Interest

The authors declare no conflicts of interest.

## Data Availability

The data that support the findings of this study are available from the corresponding author upon reasonable request.
